# An increase of CD83^+^ dendritic cells *ex vivo* correlates with increased regulatory T cells in patients with active eosinophilic granulomatosis and polyangiitis

**DOI:** 10.1186/s12865-014-0032-5

**Published:** 2014-08-31

**Authors:** Naomi Tsurikisawa, Hiroshi Saito, Chiyako Oshikata, Takahiro Tsuburai, Miyako Ishiyama, Hiroyuki Mitomi, Kazuo Akiyama

**Affiliations:** Departments of Allergy and Respirology, 18-1 Sakuradai, Minami-ku Sagamihara, Kanagawa 252-0392 Japan; Clinical Research Center for Allergy and Rheumatology, National Hospital Organization, Sagamihara National Hospital, 18-1 Sakuradai, Minami-ku Sagamihara, Kanagawa 252-0392 Japan; Laboratory Medicine, Kanagawa Rehabilitation Hospital, 516 Nanasawa Atsugi, Kanagawa, 243-0121 Japan; Department of Surgical and Molecular Pathology, Dokkyo Medical University, 880 Kitakobayashi, Shimotsuga-gun, Mibu-machi, Tochigi 321-0293 Japan

**Keywords:** Churg–Strauss syndrome, Dendritic cell, Eosinophilic granulomatosis with polyangiitis, EGPA, Regulatory T cell

## Abstract

**Background:**

Eosinophilic granulomatosis with polyangiitis (EGPA) is a rare disease characterized by the presence of allergic granulomatosis and necrotizing vasculitis with eosinophilic infiltration. The etiology of EGPA is unknown. Dendritic cells (DCs) are not only critical for the induction of primary immune responses; they may also be important for the induction of immunological tolerance and the regulation of the type of T-cell-mediated immune response. To investigate whether DC maturation is associated with EGPA disease status, we examined the relationship between the maturation of DCs and the differentiation of regulatory T (T_reg_) cells in EGPA patients. We exposed the CD14^+^ blood monocytes of 19 patients with EGPA in remission or relapse to stimulation with GM-CSF and IL-4 for 6 d and lipopolysaccharide for 24 h to obtain mature CD83^+^ DCs and immature CD206^+^ DCs. Using immunohistochemistry, we examined four patients for the presence of CD83^+^ and CD206^+^ DCs in the lung at the onset of EGPA.

**Results:**

The percentage of CD83^+^ cells among DCs differentiated from CD14^+^ monocytes was lower for EGPA patients in relapse than in remission. The percentage of CD83^+^ DCs was inversely correlated with the percentage of CD206^+^ DCs and was significantly correlated with the numbers of naturally occurring CD4^+^ regulatory T_reg_ (nT_reg_; FOXP3^+^CD4^+^) cells and inducible Treg (iTreg; CD4^+^CD25^+^ T cells producing IL-10 or TGF-β) cells but not the number of eosinophils. The percentage of CD206^+^ DCs was significantly inversely correlated with the percentages of nT_reg_ and iTreg cells but not the number of eosinophils. Immunohistochemistry revealed both CD206^+^ DCs and CD83^+^ DCs in alveoli and interstitial spaces at the onset of EGPA.

**Conclusion:**

The maturation of DCs from monocytes was related to disease activity in patients with EGPA. Increased CD83^+^ DCs in EGPA patients may induce the differentiation of iT_reg_ and nT_reg_ cells, thereby suppressing inflammation and disease activity.

## Background

Eosinophilic granulomatosis with polyangiitis (EGPA; also known as Churg–Strauss syndrome) is a rare disease characterized by the presence of allergic granulomatosis and necrotizing vasculitis. Development of the condition is preceded by peripheral blood eosinophilia and eosinophilic tissue infiltration [1]. EGPA is classified as a vasculitis of arteries that are small or medium in diameter, although such vasculitis often is not apparent in the initial phases of the disease [2]. Asthma is present in 96% to 100% of EGPA patients and is the cardinal feature of EGPA. Asthma may precede systemic vasculitis by approximately 8 years and, in some cases, by more than 30 years [1,3,4]. The precise etiologies of EGPA and the asthmatic phase that precedes it, as well as the role of genetic factors, remain unknown. In our retrospective cohort study [5], we found that most patients in the prevasculitic phase of EGPA had severe asthma requiring treatment with systemic steroids or mechanical ventilation, sinusitis, and a high percentage of eosinophils in the peripheral blood. Chronic eosinophilic pneumonia precedes systemic vasculitis in half of all patients with EGPA [4,6].

Regulatory T (T_reg_) cells play central roles in the maintenance of both immune homeostasis and peripheral tolerance. Naturally occurring CD4^+^ T_reg_ (nT_reg_) cells that constitutively express the forkhead box P (FOXP) 3 protein can also be identified because of their expression of cytotoxic T-lymphocyte antigen-4 (CTLA-4), CD25, and CD4 [7]. Peripheral differentiation of inducible T_reg_ (iT_reg_) cells form naïve CD4 T cells, which secrete IL-10, requires an elevated level of serum TGF-β and increased of IL-10 [8]. We previously confirmed that the maintenance of T_reg_ cell numbers in asthma patients with chronic eosinophilic pneumonia may inhibit EGPA development via the action of cytokines, such as IL-10 and TGF-β, which are produced by CD4^+^CD25^+^ cells, and IL-2, which is produced by CD4^+^CD25^−^ T cells [9].

The mainstay of treatment for EGPA is systemic corticosteroid therapy. Additional treatment with immunosuppressive agents such as cyclophosphamide, azathioprine, methotrexate, mycophenolate mofetil, rituximab, interferon-α, anti-IgE antibodies, anti-IL-5 antibodies, anti-TNF-α (infliximab, etanercept, adalimumab), plasma exchange, and intravenous immunoglobulin therapy (IVIG) may be of benefit to some patients [10]. The relapse rate is higher among patients with EGPA than with microscopic polyangiitis (MPA); some patients with EGPA experience frequent relapses after initial clinical remission, whereas others fail to achieve remission that lasts for an extended period [11]. The mechanisms underlying the intractable form of EGPA remain poorly understood.

In our previous studies, frequently relapsing EGPA patients, defined as patients who relapse at least once every 2 years after an initial period of remission, had decreased numbers of T_reg_ cell counts, CD19^+^ B cells, and a lower serum IgG concentration than those who did not experience frequent relapses. In patients with frequently relapsing EGPA, decreases in T_reg_ cell numbers and increased percentages of activated B cells, such as CD80^+^, CD27^+^, and CD95^+^ B cells, may induce apoptosis of B cells [12]. T_reg_ cells are involved in the etiology and mechanisms of EGPA, but their precise role is unknown, especially in relation to antigen-presenting cells such as dendritic cells (DCs).

DCs are widely recognized as the strongest antigen-presenting cells and have the unique ability to induce a primary immune response [13]. DCs can prevent or inhibit T-cell-mediated effector responses through a variety of mechanisms, ranging from the production of pleiotropic anti-inflammatory factors that exert broadly attenuating effects to the induction of antigen-specific T cell responses that result in anergy, deletion, or instruction of T_reg_ cells [14,15]. DCs bind to T_reg_ cells in the presence of the CD80/86 co-stimulatory molecule [14]. Immature DCs induce IL-10–producing iT_reg_ cells, reinforcing peripheral tolerance [16].

Conventional DCs (cDCs, also known as myeloid DCs) may play a pathophysiological role in the inflammation associated with antineutrophil cytoplasmic autoantibody (ANCA)-associated vasculitis [17–19], granulomatosis polyangiitis–Wegener granulomatosis [20], EGPA [21], Kawasaki disease [22], and giant cell arteritis (GCA) [23]. Local increases in the number of activated myeloid DCs probably impede the maintenance of tolerance and lead to vascular autoimmune inflammation [24].

CD83 is a cell-surface marker characteristic of fully mature DCs [25–27]. In their function as antigen-presenting cells, CD83-positive DCs induce T-cell-mediated immune responses [26]. CD83-positive DCs have been associated with activated EGPA [21] and multiple sclerosis [28]. Injections of soluble CD83 clearly reduce paralytic symptoms in murine experimental autoimmune encephalomyelitis [29]. CD206 (macrophage mannose receptor, C type I) antigen is upregulated in M2-type macrophages [30]. CD206-positive antigen-presenting cells have high phagocytic capacity. CD206 is expressed on immature DCs generated from monocytes (MoDCs), whereas CD83 is expressed on mature MoDCs [31].

Although some reports suggest an association between DCs and T_reg_ cells [18,32,33], the details are still unclear. To further explore whether DCs or T_reg_ cells play a role in the remission or relapse of EGPA, we examined the relationship between the maturation of DCs and the differentiation of T_reg_ cells in EGPA patients.

## Results

### Clinical findings and treatment

Nineteen patients with EGPA were enrolled in the study (Table 1). All patients had adult onset of the disease and received systemic corticosteroids; 12 of the 19 patients had immunosuppressants during initial treatment. Blood was examined in 12 of the 19 patients at relapse and in 14 of the 19 patients at remission. The Birmingham Vasculitis Activity Score was higher in patients at relapse than at remission. A higher percentage of patients used immunosuppressants at relapse than at remission (Table 2). The number of eosinophils in peripheral blood did not differ between the two groups, but the percentage of FOXP3^+^ cells among CD4^+^ T cells (*P* < 0.01) and the percentages of CD4^+^CD25^+^ T cells positive for IL-10 (*P* < 0.01), TGF-β (*P* < 0.01), and IL-2 (*P* < 0.05; Mann–Whitney U test) were lower in patients at relapse than in those at remission (Table 2).

**Table 1:** **Characteristics and therapy of patients with EGPA (** ***n*** **= 19)**

Age at the time of the study (years)	58.9 ± 16.9*
Male/Female	7/12
Atopic/nonatopic	6/13
Age at onset of EGPA (years)	52.6 ± 18.4
Duration of EGPA (years)	6.8 ± 3.6
At onset of EGPA	
WBCs (/μL)	15561 ± 6238
Eosinophils (/μL)	8344 ± 5887
MPO-ANCA positive/negative	6/13
Clinical manifestations of EGPA	
Number of organs involved	5.4 ± 1.9
Five-Factor Score 1996	1.1 ± 0.8
Five-Factor Score 2009	1.4 ± 0.9
Additional organ involvement	
Asthma (yes/no)	19/0
Sinusitis (yes/no)	18/1
Lung (yes/no)	11/6
Multiple mononeuritis (yes/no)	19/0
Lowest MMT score	3.2 ± 1.1
Heart (yes/no)	14/5
Gastrointestinal (yes/no)	16/3
Renal (yes/no)	7/10
Skin (yes/no)	12/5
Central nervous system (yes/no)	4/15
Arthralgia (yes/no)	9/10
Myalgia (yes/no)	7/10
BVAS at onset	35.1 ± 10.1
Initial treatment	
Initial dose of PSL (mg)	44.2 ± 10.7
Immunosuppressant at initial treatment (yes/no)	12/7
CYC/AZA/CSA/MTX/RTX	10/1/1/0/0

WBC, white blood cell; MPO-ANCA, myeloperoxidase-specific antineutrophil cytoplasmic autoantibody; MMT, the manual muscle test; BVAS, Birmingham Vasculitis Activity Score; PSL, prednisolone; AZA, azathioprine; CSA, cyclosporin A; CYC, cyclophosphamide; MTX, methotrexate; RTX, rituximab.

*Values are given as mean ± 1 standard deviation or as number.

**Table 2:** **Patient characteristics at assay**

	**Relapse** ***n*** **= 12**	**Remission** ***n*** **= 14**	***P***
Duration from onset of EGPA (months)	61.3 ± 100.7	75.1 ± 96.4	NS^*^
BVAS at relapse or remission	14.5 ± 9.5	0 ± 0	< 0.01^*^
Prednisolone dose at relapse or remission	10.4 ± 4.6	9.6 ± 7.6	NS^*^
Immunosuppressant use at relapse or remission (%)	75	35.7	<0.05
CYC/AZA/CSA/MTX/RTX	7/1/1/0/0	3/2/0/0/0	NS^‡^
No. of eosinophils in peripheral blood	350.8 ± 262.0	309.3 ± 273.2	NS^*^
FOXP3^+^ cells among CD4^+^ T cells (%)	0.5 ± 0.4	5.8 ± 2.1	< 0.01^†^
IL-10^+^ cells among CD4^+^CD25^+^ T cells (%)	0.3 ± 0.8	10.9 ± 11.4	< 0.01^†^
TGF-β^+^ among CD4^+^CD25^+^ T cells (%)	0.2 ± 0.7	7.4 ± 6.1	< 0.01^†^
IL-2^+^ among CD4^+^CD25^−^ T cells (%)	9.9 ± 14.1	32.2 ± 25.3	< 0.05^†^

BVAS, Birmingham Vasculitis Activity Score; AZA, azathioprine; CSA, cyclosporin A; CYC, cyclophosphamide; MTX, methotrexate; RTX, rituximab; NS, not significant.

A *P* value of <0.05 was considered to be statistically significant.

^*^Two-way ANOVA using repeated-measures algorithm.

^†^Mann–Whitney U-test.

^‡^Chi-squared testing revealed no significant differences between the frequencies of the characteristics measured in the two groups.

### Expression of CD83 and CD206 in CD14^+^ monocytes that differentiated into DCs

**Figure 1:**
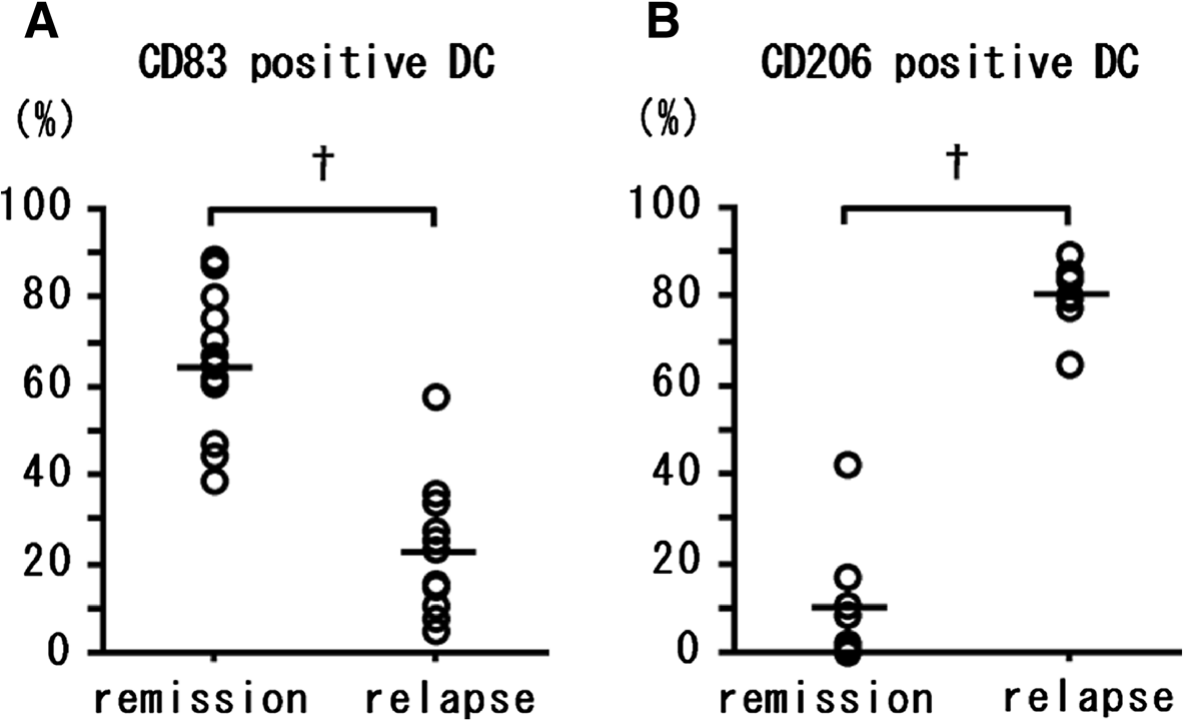
**Mature and immature DCs in EGPA patients during remission and relapse.** DC cells were induced from monocytes in peripheral blood cells from patients with EGPA during remission or relapse, and cell-surface markers were labeled by using immunocytochemistry. **(A)** Percentage of DCs positive for CD83 (mature DCs). **(B)** Percentage of cells positive for CD206 (immature DCs). Differences between patient groups were evaluated by using the Mann–Whitney U test. †, *P* < 0.01.

**Figure 2:**
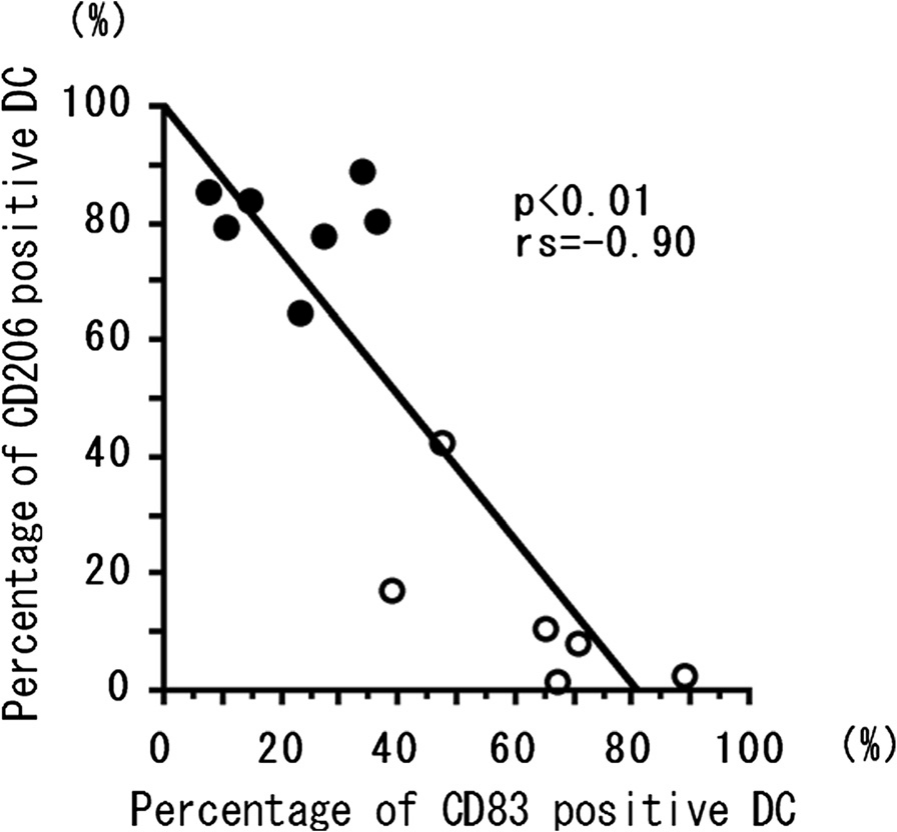
**Inverse correlation between percentages of mature and immature DCs.** Correlation between the percentages of CD83-positive DCs and CD206-positive DCs induced from monocytes in peripheral blood cells in EGPA patients experiencing relapse (*closed circles*) and remission (*open circles*). The correlation coefficient (rs) was obtained by using the Spearman’s rank correlation test.

The percentage of CD83^+^ cells among MoDCs was 28.4% ± 15.7% in healthy subjects compared with 64.6% ± 15.7% in EGPA patients at remission and 22.1% ± 15.3% in patients with EGPA at relapse. The percentage of CD206^+^ cells among MoDCs was 16.65 ± 7.1% in healthy subjects, 10.3% ± 14.3% in patients whose EGPA was in remission, and 80.0% ± 7.8% in patients with EGPA at relapse. The percentage of CD83^+^ cells among MoDCs was lower in EGPA patients in relapse than in remission (*P* < 0.01, Mann–Whitney U test; Figure 1A). In contrast, the percentage of CD206^+^ cells among MoDCs was higher in EGPA patients during relapse than remission (*P* < 0.01, Mann–Whitney U test; Figure 1B); technical difficulties precluded the assessment of the percentage of CD206^+^ cells in 6 of the 19 patients. The percentage of CD83^+^ DCs was inversely correlated with the percentage of CD206^+^ DCs (*P* < 0.01, Spearman’s rank correlation test; Figure 2). We confirmed that the percentage of single-labelled CD83^+^ DCs was significantly correlated with that of CD83^+^ DCs with the CD11c^+^CD19^−^ (*P < 0.01*, rs = 0.89) or CD11c^+^CD56^−^ (*P < 0.01*, rs = 0.99) expression patterns.

### Correlation of CD83^+^ and CD206^+^ DCs and T_reg_ cells

**Figure 3:**
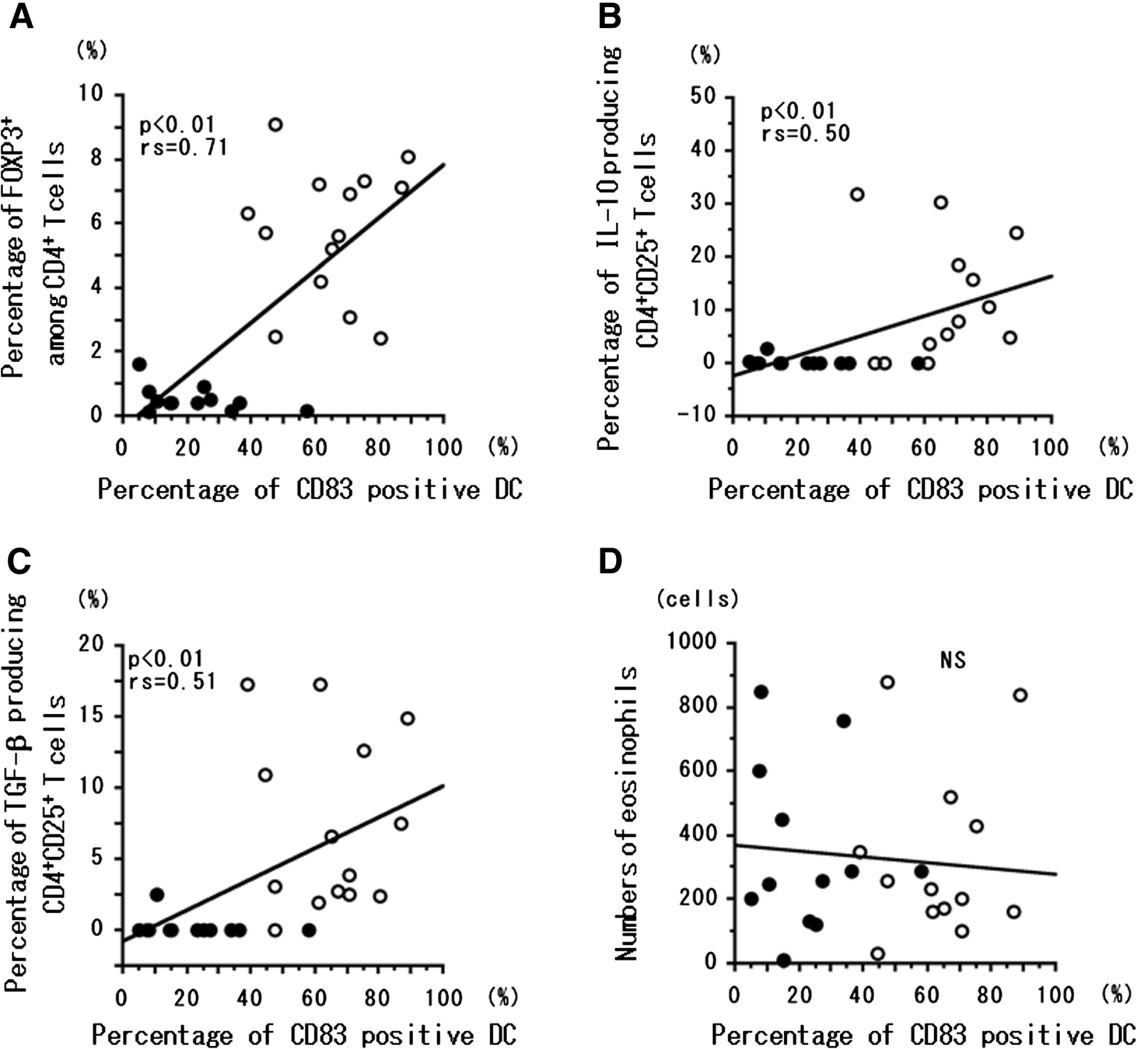
**Correlation of mature DCs with other immune cell types.** Correlation between the percentage of CD83-positive DCs and the percentages of FOXP3^+^ CD4^+^ T (nT_reg_) cells **(A)**, IL-10^+^ CD25^+^CD4^+^ T (iT_reg_) cells **(B)**, and TGF-β^+^ CD25^+^CD4^+^ T (iT_reg_) cells **(C)** and the number of eosinophils **(D)** in peripheral blood. Correlation coefficients (rs) were obtained by using Spearman’s rank correlation test. Closed circles, patients experiencing relapse; open circles, patients in remission.

**Figure 4:**
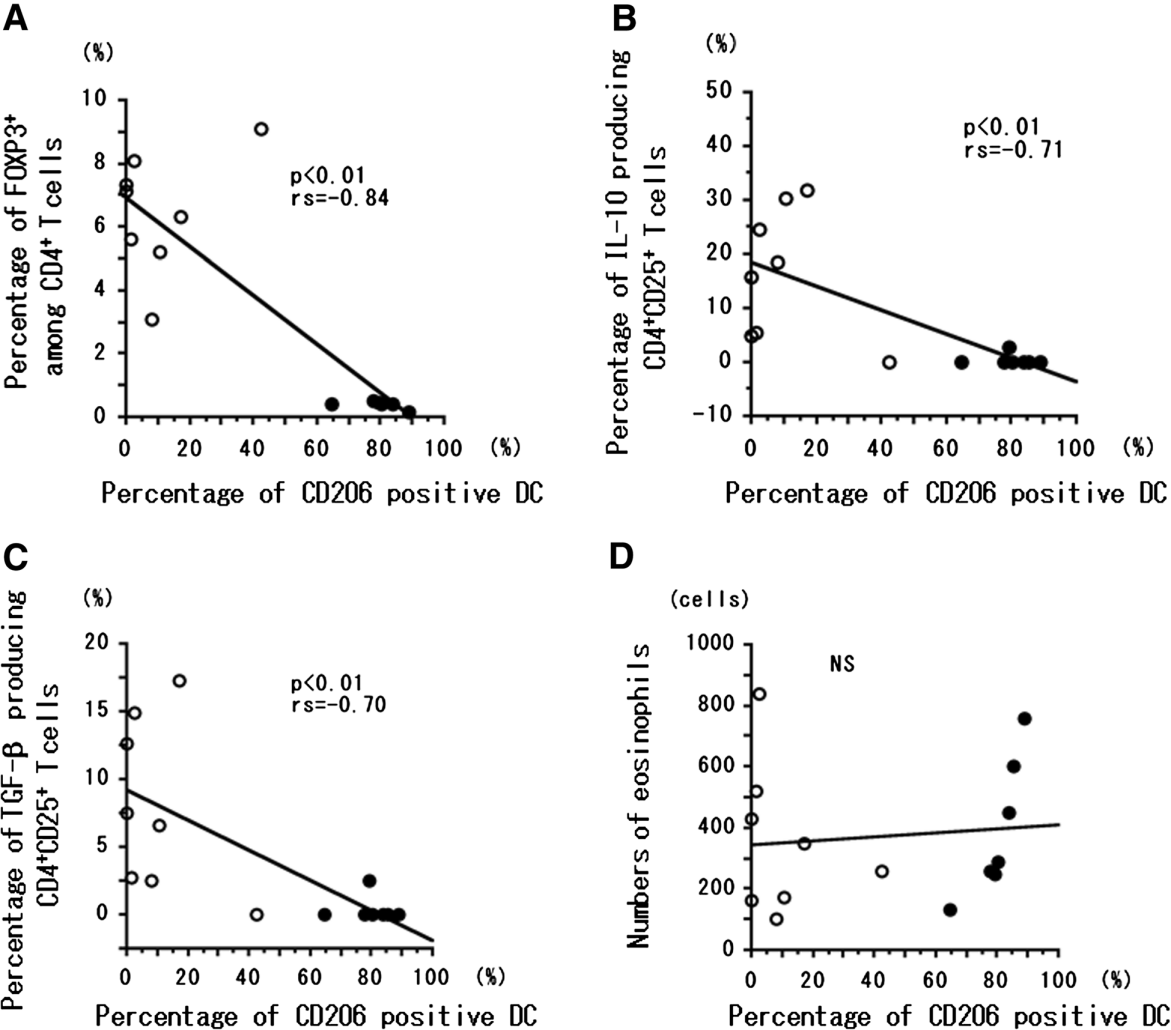
**Correlation of immature DCs with other immune cell types.** Correlation between percentage of CD206^+^ DCs and the percentages of FOXP3^+^ CD4^+^ T cells **(A)**, IL-10^+^ CD25^+^CD4^+^ T cells **(B)**, and TGF-β^+^ CD25^+^CD4^+^ T cells **(C)** and the number of eosinophils **(D)** in peripheral blood. Correlation coefficients (rs) were obtained by using Spearman’s rank correlation test. Closed circles, patients experiencing relapse; open circles, patients experiencing remission.

**Figure 5:**
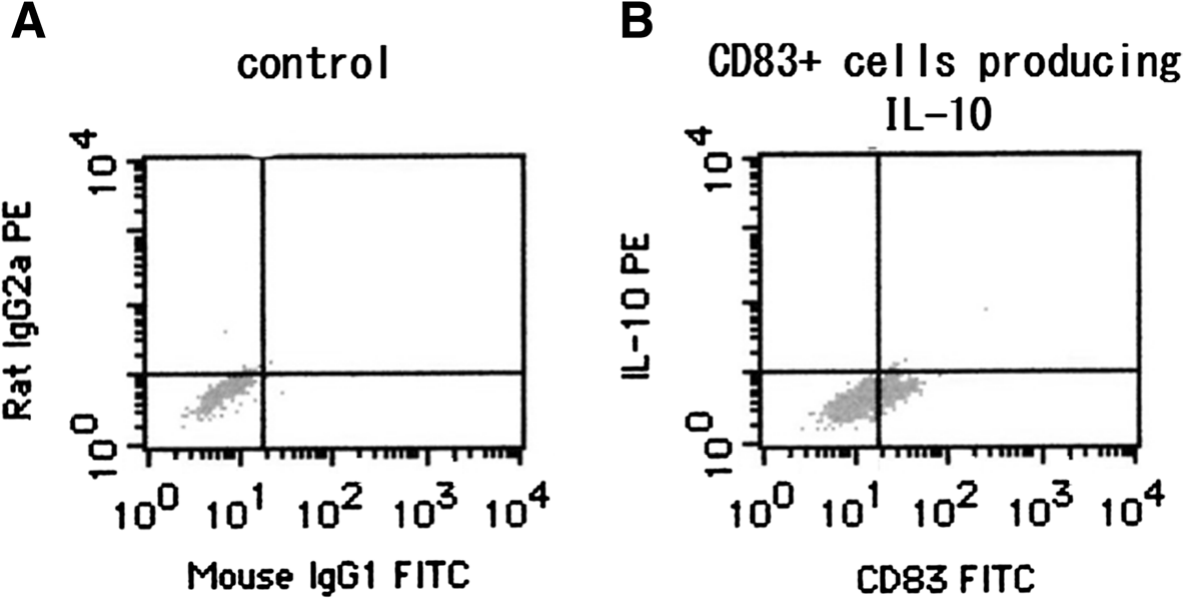
**CD83** ^**+**^ **DC producing IL-10.** CD83-positive DCs treated with a control **(A)** or an anti-IL-10 antibody **(B)** after CD14^+^ monocytes from patients with EGPA in remission were incubated with GM-CSF and IL-4, stimulated with lipopolysaccharide, and analyzed by flow cytometry.

The percentage of CD83^+^ DCs was significantly (*P* < 0.01) correlated with the percentages of FOXP3^+^ CD4 T cells (Figure 3A), CD4^+^CD25^+^ T cells producing IL-10 (Figure 3B), and CD4^+^CD25^+^ T cells producing TGF-β (Figure 3C). However, the percentage of CD83^+^ DCs was not correlated with the number of eosinophils in peripheral blood (Figure 3D). In contrast, the percentage of CD206^+^ DCs was significantly (*P* < 0.01) inversely correlated with the percentages of FOXP3^+^ CD4^+^ T cells (Figure 4A), CD4^+^CD25^+^ T cells producing IL-10 (Figure 4B), and CD4^+^CD25^+^ T cells producing TGF-β (Figure 4C). The percentage of CD206^+^ cells among DCs was not correlated with the number of eosinophils in peripheral blood (Figure 4D). We confirmed that CD83^+^ DCs induced from CD14^+^ monocytes produced IL-10 in patients with EGPA at remission (Figure 5). In the three patients of this study with EGPA at remission, the percentage (mean ± SD) of CD83^+^DCs that produced IL-10 was 0.76% ± 0.73%.

### Immunochemistry of CD83^+^ and CD206^+^ DCs *ex vivo* and *in vivo*

**Figure 6:**
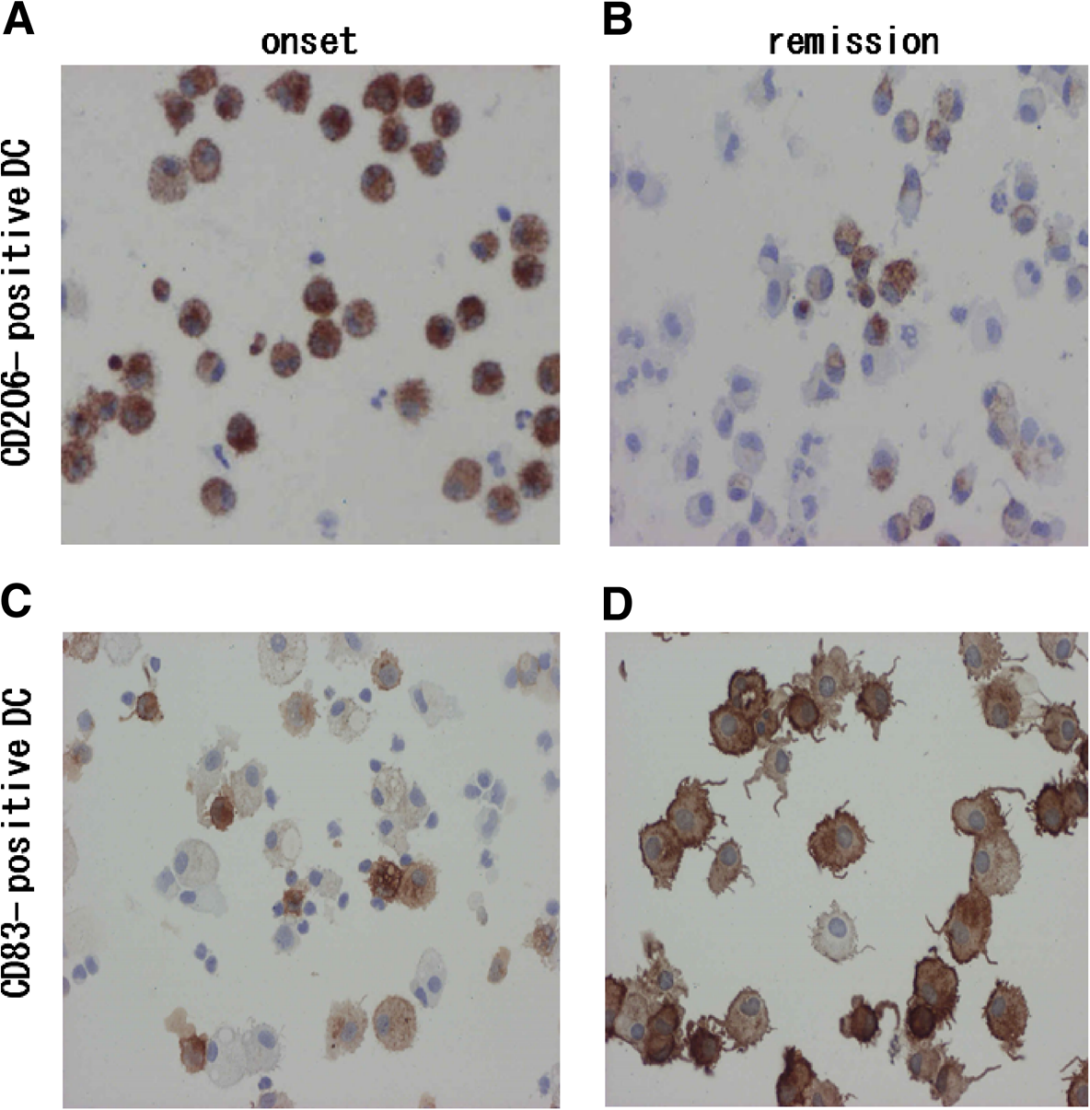
**Mature and immature DCs at onset and remission.** DCs induced from monocytes in peripheral blood of patients with EGPA at onset **(A, C)** or remission **(B, D)** were stained for CD83 **(A, B)** or CD206 **(C, D)**. Magnification, ×200.

We used an anti-CD206 antibody to label immature MoDCs obtained from patients with EGPA. Immature CD206^+^ DCs had few dendrites and a rounded form (Figure 6A). More CD206^+^ immature DCs were present in patients with EGPA at onset than at remission (Figure 6 A,B). In contrast, mature DCs labeled with an anti-CD83 antibody had many dendrites (Figure 6D) and were more frequent in patients with EGPA in remission than in those at EGPA onset (Figure 6C,D).

**Figure 7:**
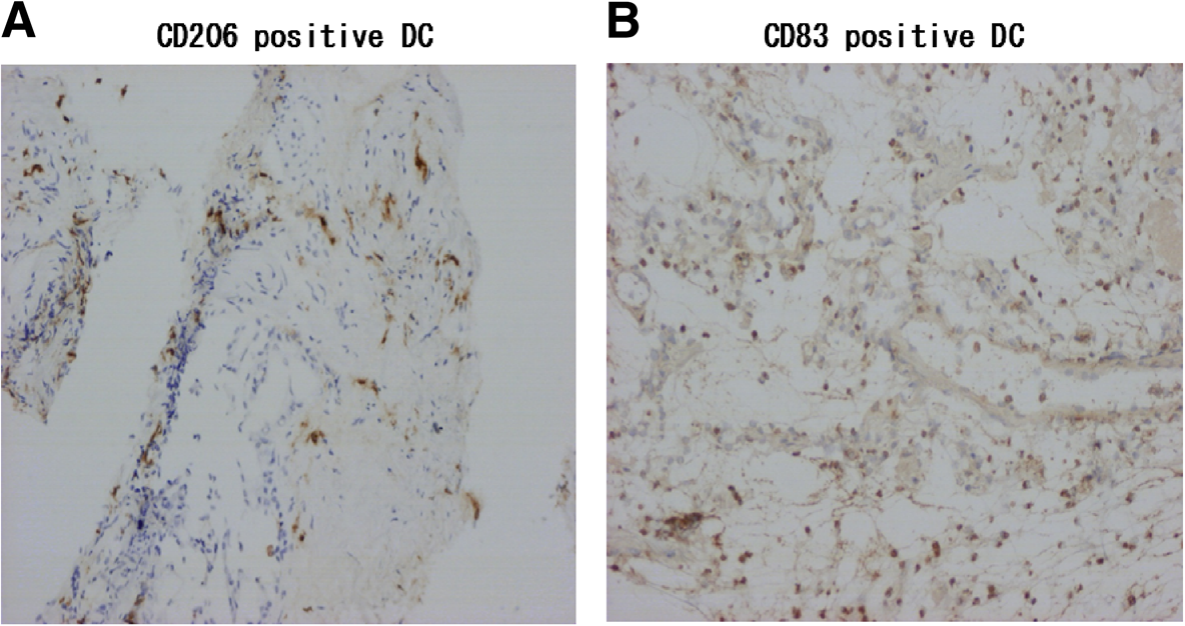
**DCs in a lung biopsy.** Immunohistochemistry for CD206 **(A)** and CD83 **(B)** was performed on lung tissue from a representative patient with EGPA at onset. Magnification × 100.

We labeled CD206^+^ immature and CD83^+^ mature DCs of five patients of 19 patients who had ground-glass opacity or consolidation in the lungs. Eleven of 19 patients had lung involvements by diagnostic imaging. Lung biopsy tissue from a representative five patients with EGPA at onset who had not been treated with systemic corticosteroids had both CD206^+^ DCs (Figure 7A) and CD83^+^ DCs (Figure 7B) in the alveoli and interalveolar spaces. CD83^+^ DCs also were present in the respiratory bronchioles. However, we saw few CD83^+^ or CD206^+^ DCs in lung tissue from EGPA patients at remission after treatment with corticosteroids, immunosuppressants, and IVIG (data not shown).

## Discussion

Not only are DCs crucial for the induction of primary immune responses, but these cells may also be important for the induction of immunological tolerance and the regulation of the type of T-cell–mediated immune response [34]. The cytokine Flt3 ligand and its receptor Flt3required for the development of plasmacytoid DCs, and cDCs [35]. Plasmacytoid DCs develop in the bone marrow and accumulate mainly in the blood and lymphoid tissues and enter lymph nodes through the blood circulation [36]. Like plasmacytoid DCs, pre-cDCs develop from a common DC progenitor in the bone marrow; pre-cDCs differentiate into cDCs, which migrate through the blood to lymphoid and non-lymphoid tissues [37]. Whereas plasmacytoid DCs differentiate into immunogenic DCs that can prime T cells against viral antigens [38], cDCs have an enhanced ability to sense and respond to tissue injury, capture environmental and cell-associated antigens, process and present phagocytosed antigens to T lymphocytes, and prime T cell responses [37].

CD83^+^ DCs have been reported to reflect disease activity in inflammatory disease [21,28,29]. Here we have shown *in vivo* that both mature CD83^+^ DCs and immature CD206^+^ DCs can be present in the alveoli and interstitial spaces of the lungs at the onset of EGPA. After we induced the differentiation of patient-derived MoDCs *ex vivo*, we found that MoDCs that expressed CD83^+^ were predominant in patients in remission, whereas MoDCs expressing CD206^+^ predominated in samples from patients at relapse. Many kinds of inflammatory cells, including eosinophils, effector T cells, T_reg_ cells, Th1 cells, Th2 cells, and Th17 cells, are present in the peripheral blood during the active phase of EGPA [39,40]. We hypothesized that the inflammatory cells present in increased numbers during the active phase might migrate into the alveoli and interstitial spaces of the lungs.

In patients with EGPA, the number and percentage of mature CD83^+^ DCs were inversely correlated with those of immature CD206^+^ MoDCs. In addition, the percentage of CD83^+^ DCs was higher in patients with EGPA in remission than in relapse and correlated with the percentage of T_reg_ cells. According to previous reports [18,33], CD83^+^ DCs suppress the immune response, probably by inducing T_reg_. Mature DCs expressing CD83^+^ at remission, which were present in higher numbers after treatment with corticosteroids, immunosuppressants, and IVIG, might induce the differentiation of both iT_reg_ cells and nT_reg_ cells. In contrast, we saw few CD83^+^ or CD206^+^ DCs in the lung *in vivo* at remission after treatment with corticosteroids, immunosuppressants, and IVIG, but this therapy did not affect the number of eosinophils, which is already high in patients with EGPA before the onset of vasculitis.

We previously reported that the percentage of CD4^+^ T cells producing IL-17 and IL-22 (Th17 cells) is significantly greater in patients with active EGPA than in healthy controls or in patients with asthma, chronic eosinophilic pneumonia, or inactive EGPA [40]. As stated earlier, eosinophilia is characteristic of EGPA, and some reports suggest that the IL-25 produced by eosinophils promotes innate adaptive immunity by enhancing Th2 cytokine production [41]. We have demonstrated that Th17 cells are correlated with the number of CD4^+^ T cells that produce IL-25 [42]. The number of Th17 cells is higher and that of iT_reg_ cells is lower in patients with active EGPA than in those with inactive EGPA [40]. The mechanism of EGPA is thought to involve the actions of Th2 cells, T_reg_ cells, and Th17 cells. This result may indicate again that mature DCs that express CD83^+^ are associated with the differentiation of T_reg_ cells but with not the number of eosinophils.

Indoleamine 2,3-dioxygenase (IDO) can be induced in many human cell types *in vitro* by stimulation with interferons, lipopolysaccharide, tumor-necrosis factor-α (TNF-α), Toll-like receptor (TLR) ligands, or FcεRI. IDO is an interferon-inducible enzyme that suppresses adaptive T-cell immunity by catabolizing the essential amino acid tryptophan in the cellular microenvironment [43]. IDO expression in monocytes from EGPA patients is positively correlated with the percentage of CD4^+^CD25^+^ T_reg_ cells producing IL-10 and inversely correlated with the percentage of Th17 cells [42]. Therefore, EGPA relapse may be linked to elevated levels of IL-25–producing CD4^+^ T cells, which promote Th2 inflammation and decrease iT_reg_ cell subpopulations, as does decreased IDO expression in monocytes. Therefore, the percentages of Th17 cells and of CD4^+^CD25^+^ T_reg_ cells producing IL-10 both reflect the disease activity of EGPA [42].

Together with co-stimulatory molecules such as CD80 and CD86, CD83 is strongly upregulated during inflammation [44]. Ligation of CTLA-4 to CD80 or CD86 or both may trigger the IDO pathway in DCs, in turn activating the transcription factor FOXP3 (which regulates immune functioning) and inhibiting cytokine production by DCs [45]. We demonstrated that patients with EGPA who experience frequent relapses after initial clinical remission have decreased T_reg_ cell counts; increased percentages of B cells positive for CD80, CD27, or CD95; lower CD19^+^ B cell counts; and lower serum IgG than do those who maintain remission [12]. In contrast, other patients never achieve clinical remission [12]. These results suggest that an interaction between T_reg_ cells and B cells via the overexpression of costimulatory molecules such as CD80 is related to EGPA disease severity.

TLRs constitute a family of transmembrane proteins that are expressed by various cell types, including antigen-presenting cells. TLRs function to discriminate pathogens and initiate inflammatory signaling pathways [46]. TLR4, TLR5, TLR7, and TLR9 may play a role in vasculitis [47,48]. DCs localized at the adventitia–media border of medium-sized arteries in patients with GCA express a series of TLR receptors, produce chemokines, and activate T cells [49]. LPS-induced triggering of TLR4 activates DCs, suggesting a key role for TLR4 in the induction of the inflammatory processes in GCA [50]. These researches may help to elucidate the etiology of vasculitis.

The remission rate after initial treatment is reported to be 81% to 91% in EGPA patients, compared with 30% to 93% in patients with GPA and 75% to 89% in those with MPA [11]. However, the relapse rate during the first 2 years after diagnosis is higher in EGPA patients (35%) than in MPA patients (8%) [11]. The relapse rate at 5 years or more after disease onset is greater than 60% in patients with EGPA, compared with 20% in MPA patients [51]. Why the relapse rate of EGPA is so high is unknown, but vasculitis relapse might occur when unknown events in the peripheral blood or blood vessels lead to an interaction between DCs and T_reg_ cells, thereby causing an immune response.

## Conclusion

We conclude that CD83^+^ DCs are related to EGPA disease activity. Specifically, an increase in the number of CD83^+^ DCs generated from monocytes may induce the differentiation of iT_reg_ and nT_reg_ cells and subsequently lead to remission in patients with EGPA.

## Methods

### Patients

Between March and October 2006, we recruited 19 patients with EGPA and 7 healthy subjects at the Clinical Research Center for Allergy and Rheumatology (National Hospital Organization, Sagamihara, Kanagawa, Japan). The Ethics Committee of our hospital approved the study, and written informed consent was obtained from each subject. EGPA was defined in all patients according to the classification criteria of the American College of Rheumatology [52]. A patient was considered to be in remission when the disease was inactive and when no clinical sign or symptom of active vasculitis had been evident for 6 months or more. A relapse was defined as the presence of active disease combined with the recurrence, after initial remission, of vasculitis symptoms (with or without an increase in the percentage of eosinophils among white blood cells). Patients who relapsed required resumption of immunosuppressive therapy or increased doses of immunosuppressant.

Disease activity was assessed at first diagnosis and at first relapse by using the Birmingham Vasculitis Activity Score [53]. This scheme evaluates symptoms and signs within 9 categories (systemic; cutaneous; mucous membranes and eyes; ear, nose, and throat; chest; heart and vessels; gastrointestinal tract; renal system; and nervous system). The maximal number of possible points in each category is 7; the maximal score is thus 63. In addition, blood samples obtained from patients with EGPA during relapse (in the active disease state) or remission (when the disease was inactive) were evaluated.

Multiple mononeuritis, a measure of motor nerve dysfunction, was evaluated by using the manual muscle test; responses were scored from 0 to 5 on the Medical Research Council scale. Sensory nerve dysfunction was evaluated by clinical examination. Lung involvement was considered present when any of the following was present: consolidation, ground-glass opacity, nodules within such opacity, interlobular septal thickening, bronchial wall thickening, lymph node enlargement, pleural effusion evident upon high-resolution computed tomography, or eosinophilic infiltration detected by lung biopsy. The heart was considered to be involved when any of the following was evident: chest pain, chest discomfort, back pain, palpitations, abnormal signs on cardiac echocardiography, Holter electrocardiographic abnormalities, elevated B-type natriuretic peptide levels, or [^123^I]-meta-iodobenzylguanidine myocardial imaging abnormalities [54]. Gastrointestinal involvement was indicated by the presence of symptoms of epigastralgia, abdominal pain, diarrhea, constipation, or positive endoscopic signs, combined with confirmation of eosinophil infiltration by biopsy. Skin involvement was defined as the presence of purpura, erythema, livedo, an ulcer, or acrocyanosis when a nodule, accompanied by eosinophilic infiltration, was detected by biopsy. Central nervous system involvement was defined as the presence of headache, visual disorder, abnormal visual sensation, cerebral infarction, bleeding, or cranial nerve dysfunction. Renal involvement was defined as the presence of eosinophils in urine, glomerular nephritis, nephrosis, renal dysfunction (i.e., the creatinine level was elevated more than 20% over the baseline value), or proteinuria (>0.5 g per day). Disease severity in all EGPA patients was evaluated by using the Five-Factor Score 1996 [55] or Five-Factor Score 2009 [56]. The organs compromised by asthma and sinusitis were not included in the total count of involved organs.

The eosinophils in whole blood and T_reg_ cells in peripheral blood (FOXP3^*+*^ cells among CD4^+^ T cells, or IL-10-producing cells among CD4^+^/CD25^+^ T cells, or TGF-β-producing cells among CD4^+^/CD25^+^ T cells) were counted in all patients at relapse or during remission.

### Immunological analysis

#### Differentiation of DCs expressing CD83^+^ or CD206^+^ from CD14^+^ blood monocytes

Mononuclear cells were isolated by using Ficoll–Paque (Lymphocyte Separation Media, catalog no. CPL-50494-5, Cosmo Bio. Co. Ltd., Tokyo, Japan) density-gradient centrifugation of heparinized blood obtained from EGPA patients [27]. To this end, white blood cells treated with 4.5% dextran to remove red blood cells were washed twice with PBS and layered onto Lymphocyte Separation Media. After centrifugation at 800 × *g* for 20 minutes, the mononuclear-cell-enriched population was harvested, and these cells were washed twice with PBS after centrifugation at 300 × *g* for 10 minutes. Peripheral blood mononuclear cells (PBMCs) were resuspended (1 × 10^6^ cells/mL) in RPMI 1640 medium (GIBCO/BRL, Grand Island, NY, USA) containing 10% (vol/vol) fetal calf serum, 10 mM glutamine, and penicillin–streptomycin. The resulting T-cell-depleted mononuclear cells were cultured at 37°C for 3 h in 100-mm tissue culture plates and washed twice with PBS to remove residual debris from the plates. The purified plastic-adherent monocyte population was confirmed to be more than 97% CD14^+^ monocytes.

CD14^+^ monocytes were cultured in 6-well tissue culture plates (1 × 10^6^ cells/mL) in medium containing cytokines (GM-CSF [50 ng/mL] and IL-4 [10 ng/mL]) purchased from BioLegend (Cosmo Bio. Co. Ltd.) or R&D Systems (Tokyo, Japan). The cultures were fed with fresh medium and cytokines every 2 d, and cell differentiation was monitored by light microscopy as described [27]. After treatment with GM-CSF and IL-4 for 6 d, CD14^+^ monocytes were stimulated by exposure to lipopolysaccharide for 24 h by fluorescence-based cell sorting as described [57]. The collected cells were placed on silanized slides and frozen at −80°C. Sections were pre-incubated with PBS containing 5% (w/v) bovine serum albumin (BSA; Sigma-Aldrich) for 30 min, followed by incubation with mouse anti-CD83 antibody (BioLegend, Cosmo Bio Co. Ltd.) or mouse anti-CD206 antibody (BD Biosciences, Rikaken Co. Ltd., Tokyo, Japan) for 60 min [57,58]. The slides were developed by incubation with 100 μL of DAB and then lightly counterstained with Mayer’s hematoxylin. The percentage of CD83^+^ or CD206^+^ cells among CD14^+^ monocytes stimulated with GM-CSF, IL-4, and lipopolysaccharide was evaluated by using image analysis software (Image Pro Plus 6.0, Media Cybernetics, Bethesda, MD, USA).

As described earlier, the percentages of CD83^+^ DCs expressing CD11^+^, CD19^−^, or CD56^−^ expression patterns from CD14^+^ blood monocytes and the percentage of CD83^+^ DCs producing IL-10 were analyzed by flow cytometry.

### Transbronchial lung biopsy

Bronchoscopy was performed in accordance with international guidelines [59]. After premedication with 4% lidocaine by nebulizer and spray, all patients were anaesthetized intravenously with 3 mg midazolam hydrochloride. A bronchoscope (Olympus BF-1 T240, Olympus Optical Co., Tokyo, Japan) was inserted through the mouth and pharynx, and the trachea and bronchi were anaesthetized with 4% lidocaine administered during maintenance with oxygen at 3 L/min via nasal cannula. Four patients who had ground-glass opacity or consolidation on computed tomography underwent lung biopsies at onset of EGPA. Two or three specimens per patient were taken from lungs with ground-glass opacity or consolidation on computed tomography images.

### Immunohistochemistry of the lung

Lung specimens were embedded in Optimal Cutting Temperature compound (OCT, Bayer-Pharma, Zurich, Switzerland) and frozen at −80°C. We immunostained 8-μm sections on silanized slides in an Autostainer slide processor (DakoCytomation, Kyoto, Japan) as described [60]. Briefly, the sections were incubated with 10% formalin for 15 min, and then washed with PBS twice for 5 min each wash. Endogenous peroxidase activity was blocked by incubation with peroxidase-blocking solution (Dako A/S Ltd, Glostrup, Denmark) for 10 min at room temperature. The slides then were washed with PBS twice for 5 min each. Antigen retrieval for all antibody treatments consisted of autoclave treatment of sections for 30 min in either Epitope Retrieval Solution (DakoCytomation) or Target Retrieval Solution (pH 6.0, DakoCytomation). The slides were incubated with primary antibodies overnight at 4°C by using Envision kits (DakoCytomation), followed by 30 min with horseradish peroxidase–labeled complex conjugated with secondary antibody and 30 min with substrate–chromogen (DAB) solution (DakoCytomation). The sections were lightly counterstained with Mayer’s hematoxylin. Mouse monoclonal antibodies against CD83 (BioLegend, Cosmo Bio Co. Ltd.) and CD206 (BD Biosciences, Rikaken Co. Ltd.) were used. Signals corresponding to CD83^+^ or CD206^+^ cells were developed by incubation in 100 μL of DAB. Staining of CD83 indicated a mature DC, and staining of CD206 indicated an immature DC [60,61].

### Enumeration of FOXP3^+^ cells among CD4^+^ T cells

Peripheral blood cells were assayed as described by Abdulahad et al. [62] to determine the percentages of CD25^*+*^ and FOXP3^*+*^ cells among CD4^*+*^ T cells. FOXP3^*+*^ cells among CD4^*+*^ T cells (termed FOXP3^*+*^ CD4^*+*^ T cells) were identified by incubating whole-blood lymphocytes with phycoerythrin-conjugated anti-FOXP3 antibody (BD Biosciences) after cell permeabilization with 4% (v/v) formaldehyde and 0.1% (w/v) saponin. Expression of surface and intracellular markers of CD4^+^ T cells (identified by an anti-CD4 antibody from BioLegend) was analyzed by flow cytometry (FACSCalibur, Nippon Becton Dickinson, Tokyo, Japan).

### Induction of cytokine expression and staining of intracellular cytokines to identify iT_reg_ cells

To induce cytokine expression and accumulation, peripheral blood mononuclear cells (PBMCs) were stimulated for 4 h at 37°C with 10 μg/mL brefeldin A in the presence or absence of 20 ng/mL phorbol myristate acetate and 1 μg/mL ionomycin [63]. We removed cells that died after such stimulation. Separated PBMCs were stimulated with 50 ng/mL phorbol myristate acetate and 1 μg/mL ionomycin for 4 h at 37°C, and 1 × 10^6^ PBMCs were suspended in RPMI 1640 medium supplemented with 1 mL of 10% FCS. The percentage of dead cells among PBMCs treated with phorbol myristate acetate and ionomycin was calculated under a microscope after addition of 40 μL trypan blue diluent. The control was whole-blood cells diluted to the same extent in FCS-free RPMI 1640 before the addition of trypan blue. The percentage of dead cells was 7.0% ± 6.7%. Dead cells could not be distinguished from live cell by flow cytometry, but the inclusion of the dead cells did not change the statistical significance of the percentage of positive cells producing cytokines. Surface-stained whole-blood lymphocyte samples were suspended in 0.5 mL cold 4% (v/v) paraformaldehyde (used as a fixative) and incubated at room temperature for 10 min. Next, the cells were washed twice with PBS and centrifuged at 200 × *g* for 7 min. Each pellet thus obtained was suspended in 2 mL serum amyloid P buffer (0.1% [w/v] saponin, 0.05% [w/v] NaN_3_ in Hanks’s balanced salt solution). Each cell suspension was again centrifuged at 200 × *g* for 7 min, and each cell pellet was suspended in 0.1 mL serum amyloid P buffer. Cell suspensions were diluted with PBS and divided into aliquots of 10^6^ cells in 20 μL. Phycoerythrin-conjugated anti-human IL-10 and TGF-β were purchased from BioLegend (San Diego, CA) and added to each tube. All tubes were vortexed and incubated for 35 min at room temperature in the dark. The percentage of cells generating cytokines was measured by flow cytometry, and the data were analyzed by using CELLQuest software (Nippon Becton Dickinson).

Human iT_reg_ cells were defined as CD4^+^/CD25^+^ T cells that produced either IL-10 as the dominant cytokine (these cells were termed IL10^+^ CD4^+^ CD25^+^ T cells) or that predominantly produced TGF-β [7]. Human nT_reg_ cells were defined as FOXP3^+^ CD4^+^ T cells and were identified as described previously [64,65].

#### Statistical analysis

All values are expressed as mean ± 1 SD unless otherwise specified. Statistical comparisons among groups were achieved by using two-way analysis of variance with a repeated-measures algorithm followed by *post-hoc* comparisons using the Newman–Keuls test. Correlation coefficients were obtained by using Spearman’s rank correlation test. A *P* value < 0.05 was considered statistically significant. Statistical analysis was performed by using SPSS for Windows, version 20 (SPSS Inc., Chicago, IL, USA).

## Abbreviation

ANCA: Antineutrophil cytoplasmic autoantibody
CTLA-4: Cytotoxic T-lymphocyte antigen-4
cDC: Conventional DC
DC: Dendritic cell
MoDC: DC generated from monocytes
EGPA: Eosinophilic granulomatosis with polyangiitis
FOXP3: Forkhead box P
GPA: Granulomatosis polyangiitis
IDO: Indoleamine 2, 3-dioxygenase
IVIG: Intravenous immunoglobulin
MPA: Microscopic polyangiitis
PBMC: Peripheral blood mononuclear cell
SLE: Systemic lupus erythematosus
TLR: Toll-like receptor
T_reg_ cells: Regulatory T cells
